# Do sexual expectancies and inhibitions predict high-risk sexual behaviours? Evidence from a cross-sectional survey among young psychoactive substance users in informal settlements in Kampala, Uganda

**DOI:** 10.1186/s12889-021-11536-8

**Published:** 2021-08-04

**Authors:** Tonny Ssekamatte, Simon P. S. Kibira, Moses Tetui, John Bosco Isunju, Richard K. Mugambe, Solomon Tsebeni Wafula, Esther Buregyeya, Christine Kayemba Nalwadda, Justine Nnakate Bukenya, Rhoda K. Wanyenze

**Affiliations:** 1 Department of Disease Control and Environmental Health, Makerere University School of Public Health, Kampala, Uganda; 2 Department of Community Health and Behavioural Sciences, Makerere University School of Public Health, Kampala, Uganda; 3 School of Pharmacy, University of Waterloo, Waterloo, Canada; 4 Departement of Epidemiology and Global Health, Umeå University, 901 87 Umeå, Sweden; 5 Department of Health Policy, Planning and Management, Makerere University School of Public Health, Kampala, Uganda

**Keywords:** High-risk sexual behaviours, Young people, Informal settlements, Sexual expectancies, Psychoactive substance

## Abstract

**Background:**

Psychoactive substance use is a public health challenge among young people in informal settlements. Though rarely examined, psychoactive substance use is linked to sexual expectancies and inhibitions, and consequently high-risk sexual behaviours. This study examined the association between sexual expectancies and inhibitions, and high-risk sexual behaviours among young psychoactive substance users (PSUs) in informal settlements in Kampala, Uganda.

**Methods:**

This cross-sectional study recruited 744 young PSUs from informal settlements in Kampala. Respondent driven sampling was used to recruit respondents. A ‘modified’ Poisson regression model was used for inferential statistics. Data were analysed using the Stata 14 software.

**Results:**

Of the 744 study participants, 45.6% believed that psychoactive substance use improves sexual performance; 43.3% believed that psychoactive substances make sex more pleasurable, and 53.3% believed that psychoactive substances give courage or confidence to approach a partner for sex. The belief that psychoactive substance use improves sexual performance (PR 1.14, 95% CI: 1.01–1.30), increases the likelihood of engaging in sex (PR 1.20, 95% CI: 1.04–1.40) or gives courage or confidence to approach a sexual partner (PR 1.21, 95% CI: 1.05–1.39) were associated with having sex while under the influence of psychoactive substances. The belief that a psychoactive substance user under the influence of psychoactive substances is more likely to engage in sex (PR 1.48, 95% CI: 1.15–1.90), and likely to find it difficult to refuse sex (PR 1.28, 95% CI: 1.06–1.55) were positively associated with engaging in multiple sexual partnerships. The belief that one easily forgets to use a condom when under the influence of psychoactive substances was positively associated with inconsistent condom use (PR 1.26, 95% CI: 1.09–1.45).

**Conclusion:**

Psychoactive substance use expectancies associated with high-risk sexual behaviours included the belief that psychoactive substances improve sexual performance and improve confidence in approaching a sexual partner. Psychoactive substance use inhibitions associated with high-risk sexual behaviours included an increased likelihood of engaging in sexual intercourse, difficulties in refusing to engage in sexual intercourse, and forgetting to use condoms while intoxicated. Interventions targeting a reduction in high-risk sexual behaviour should integrate the impact of psychoactive substance use on sexual behaviour.

**Supplementary Information:**

The online version contains supplementary material available at 10.1186/s12889-021-11536-8.

## Background

Globally, more than a quarter of a billion people use psychoactive substances such as khat, marijuana, heroin and alcohol [1]. According to the 2020 World drug report, over 192 million people used cannabis, 58 million used opioids, 27 million used amphetamines and prescription stimulants and 19 million used cocaine [1]. The use of psychoactive substances increased at a faster rate in developing countries and was high among adolescents and young adults [1]. Data on the burden of psychoactive substance use among young people in Uganda remains scarce. However, a school survey conducted in Gulu and Kampala cities indicated that 70.1% of secondary school youths had ever used a psychoactive substance, while 39.1% were regular users of psychoactive substances [2]. The survey indicated that 20.6% of the secondary school youths in Kampala had ever used alcohol, 8.0% had ever used marijuana (*Cannabis sativa***)**, 9.9% had ever used oral tobacco (locally known as kuba), 9.7% had ever used khat (*Catha edulis*), and 9.9% had ever used aviation fuel [2]. However, the use of psychoactive substances is likely to be higher in the informal settlements due to vulnerabilities such as unemployment and poor parenting [3].

Informal settlements, defined as residences in urban areas that are characterised by limited access to social services, including those related to access to healthcare, are known to have a high prevalence of psychoactive substance use [4, 5], especially among young people [6, 7]. The high prevalence of psychoactive substance use that characterises informal settlements [6], is a predictor of high-risk sexual behaviours such as multiple sexual partnerships and unprotected sexual intercourse [8–11]. As a result, informal settlements like those in Kampala have high rates of sexually transmitted infections, including HIV [12].

The relationship between psychoactive substance use and high-risk sexual behaviours has been widely studied. Nevertheless, understanding the theoretical basis of this relationship is vital. The theoretical point of departure in this study is enshrined in the theories of alcohol myopia, cognitive escape, and expectancy. These theories provide the basis for discussions on how the use of psychoactive substances influences users’ perceptions and actions [13, 14]. Users of psychoactive substances often hold beliefs of various perceived benefits from these substances. According to the expectancy theory, users assume that psychoactive substances will reduce sexual inhibitions, such as lack of confidence in approaching sexual partners, and enhance benefits such as sexual pleasure, arousal, and performance [15–18]. Such expectancies often drive the desire to engage in high-risk sexual behaviours. Generally, a reduction in sexual inhibition and an increase in expected sexual benefits makes sexual life among users riskier [15].

Users of psychoactive substances associate them with increasing sexual arousal, delaying premature ejaculation, and prolonging sexual intercourse [16]. Such perceptions are often driven by cultural beliefs that portray good sex as long and penetrative [16]. In a society where having a sexual partner is necessary to show that someone is normal, the use of psychoactive substances has been key in stimulating erections and boosting the confidence of young people to initiate sexual relationships or engage in sexual intercourse [16].

High-risk sexual behaviours can be viewed from the perspective of impaired cognition, which can be explained by the alcohol myopia and cognitive escape theories. Based on the cognitive escape theory, individuals may opt to use certain substances to escape the self-known implications of high-risk sexual behaviours [19]. Similarly, the alcohol myopia theory points out that the use of psychoactive substances such as alcohol often alters perceptions, mood and decision making [13, 20]. Therefore, psychoactive substances are often used to disengage mental processes related to the prevention of negative consequences of high-risk sexual behaviour [20, 21]. Cognitive escape is often driven by expectancies such as better sexual pleasure and performance and is associated with turning attention away from threatening cues [22]. However, these sexual expectancies are short-lived, while the consequences of engaging in high-risk sexual behaviours pose long-term negative effects [19, 22, 23].

There is an emerging body of evidence suggesting an interaction between sexual expectancies, inhibition, and escape from self-awareness of HIV/AIDS risk and high-risk sexual behaviours, especially among men who have sex with men [21], and young people in high-income countries [24, 25]. Young people in peri-urban settings often have a higher tendency to take risks and engage in high-risk sexual encounters [26–28]. Peri-urban settings in Africa generally have a growing challenge of psychoactive substance use [29–32], but relatively less research has been conducted in these settings. Some studies also show that the use of some of the drugs such as heroin and alcohol which are viewed as less risky on the other hand often lead to injecting drug use [33–35], which is more difficult and expensive to manage [36].

The mechanisms underlying the association between psychoactive substance use and high risk sexual behaviours have been extensively examined [37]. However, there is a dearth of evidence linking psychoactive-substance use related expectancies and inhibitions, and self-reported high-risk sexual behaviours [38]. In this study, we hypothesised that sexual expectancies such as improved sexual pleasure and performance, and inhibitions are associated with high-risk sexual behaviours such as engaging in multiple sexual partnerships or relationships, inconsistent condom use, and sexual intercourse, while under the influence of psychoactive substances. Kampala Capital City was chosen as the ideal study site because of its representativeness of cities in the global south [39]. In addition, the informal settlements of Kampala are characterised by a high prevalence of psychoactive substance use and high-risk sexual behaviours among young people [40–43]. Findings of this study provide insights for targeting sexual and reproductive health interventions that aim to reduce sexually transmitted infections among young PSU in informal settlements.

## Methods

### Study design, setting and population

This was an analytical cross-sectional survey conducted between June and July 2019 among young psychoactive substance users in 12 out of the 57 informal settlements in Kampala city, Uganda. We defined an informal settlement as an urban residence characterised by inadequate access to social services and poor structural quality of housing, overcrowding and insecure residential status [44]. Such settlements are characterised by a high prevalence of psychoactive substance use [45–47] and high-risk sexual behaviours [42]. The study was conducted in four of the five divisions of Kampala (including Kawempe, Makindye, Lubaga, and Nakawa). Kampala has an estimated population of over 1.5 million people, 70% of whom (live in informal settlements [48]. Kampala is Uganda’s main economic hub, generating 65% of the national Gross domestic product (GDP).

The study participants included sexually active young people aged between 18 and 24 years who had a recent history of using at least one or more psychoactive substances, and had resided within the informal settlements of Kampala city for at least 6 months. The most commonly used and accessible psychoactive substances of interest in these settings were alcohol, *khat,* oral tobacco*,* marijuana, and heroin. The age group of 18–24 years was considered because it is a critical risk period for the initiation of substance use [1, 49]. We excluded non-residents (e.g., visiting relatives or friends), and those who were not mentally sound or intoxicated during the survey. We trained research assistants to be sensitive to behavioural signs of intoxication such as loss of co-ordination, staggering gait, drowsiness, slurred speech and glazed eyes for alcohol users, and paranoia, anxiety, eye-rolling, pupil dilation/constriction, head movements or jerks for other substances [50]. Research assistants were cautioned to remind the participants of their right to withdraw, particularly when observable signs of intoxication appeared to change [50].

### Sample size

A total of 768 young PSUs were estimated as the appropriate sample size for this survey using the Kish Leslie formula for cross-sectional studies [51]. We assumed a prevalence of high- risk sexual behaviours among young PSUs of 50% because of the limited literature in the study domain. We assumed a 5% margin of error for the estimated prevalence, 95% confidence interval around the estimates, and a design effect of 2.0 to adjust for clustering by informal settlement [52]. A design effect of 2.0 is recommended for behavioural surveys that use respondent driven sampling (RDS) [53]. A final sample of 744 young PSUs was interviewed.

### Sampling

The detailed sampling procedure for the current study has previously been reported [54]. Briefly, we purposively selected 12 informal settlements (four in Nakawa, four in Kawempe, two in Lubaga, and two in Makindye) and these were selected with the aim of geographical representativeness of informal settlements [54]. Respondent-driven sampling was used in the selection of study participants, since it is recommended for hard-to-reach populations such as young psychoactive substance users [53, 55].

During the sampling, four young PSUs were purposefully selected and enrolled as seeds from each of the 12 informal settlements, resulting in a total of 48 seeds. To ensure that the selected seeds were young psychoactive substance users, we sought guidance from peer educators, staff of non-governmental organisations working on HIV and psychoactive substance programs and the local leaders. This increased the chances of selecting seeds with a large social network. We ensured seed diversity by ensuring geographical representativeness, gender, and age group. Selecting diverse seeds was aimed at improving the chances of reaching equilibrium. Equilibrium was established when there was no significant variation in the key study variables per informal settlements i.e., age group, gender, and informal settlement.

### Data collection and study variables

Data were collected using an interviewer-administered structured questionnaire developed after a critical review of literature [56–59]. The questionnaire captured information on socio-demographic characteristics such as sex, age (in years), marital status, highest educational attainment, length of stay in a particular informal settlement, average individual monthly income, history of psychoactive substance use, sexual expectancies and inhibitions. The main outcome variables included multiple sexual partnerships, condom use and having sex under the influence of psychoactive substances. Multiple sexual partnerships were defined as having more than one sexual partner in the last 30 days [42]. Condom use was categorised as consistent and inconsistent. A respondent was considered a consistent user if he/she used condoms each time he/she had sexual intercourse with a non-spousal partner.

The main predictors in this study were sexual expectancies and inhibitions accrued from the use of psychoactive substances. Sexual expectancies were defined as perceptions of positive outcomes accrued from the use of psychoactive substances on an individual’s sexual behaviour [60]. In this study, the sexual expectancies included improved sexual pleasure or euphoria, and performance, and boosting one’s courage or confidence to approach a sexual partner. Inhibitions were defined as behaviours that go against one’s conventional or natural sexual practices [61]. Such behaviours included finding it difficult to use condoms, forgetting to use a condom, a higher likelihood of engaging in sexual intercourse, finding it difficult to decline sexual advance, and finding one’s self-wanting sex while under the influence of psychoactive substances.

The questionnaire (Additional file 1) was uploaded on the Kobo Collect application installed on android phones and tablets and interviews were conducted by well-trained data collectors. In preparation for actual data collection, the structured questionnaire (Additional file 1) was pretested among 20 young PSUs in a similar setting in Wakiso district and the feedback obtained during pretesting was used to refine the questionnaire.

### Statistical analyses

All analyses were done using Stata 14 (StataCorp, Texas, USA). We summarised categorical data using frequencies and proportions. For numerical data, we used the mean and standard deviation to provide summary statistics. We compared the substance use related perceptions on sexual expectancies and inhibitions among males and females using chi-square statistics. To establish the relationship between sexual expectancies and inhibitions, and each of the high-risk sexual behaviours, we employed robust Poisson regression with a robust error variance. We fitted three separate models in order to establish the relationship between the independent variables (sexual expectancies and inhibitions) and each of the outcome variables i.e., engaging in multiple sexual partnerships in the last 30 days, having sex while under the influence of psychoactive substances, and condom use in the last 30 days. We used prevalence ratios (PRs) as more conservative measures of association than odds ratios since the prevalence of the outcomes was greater than 10% [62]. Stepwise backward regression was used, and a cut off *p*-value of less than 0.2 was set for variables eligible to be included in the multivariable model [63]. We adjusted for known confounders such as age, sex, and level of education. A *p-*value < 0.05 was considered statistically significant.

## Results

### Characteristics of the respondents

A total of 744 young PSUs was studied. The mean age of the respondents was 21.5 years (SD ± 2.1). About 76.6% of the respondents were aged 20–24 years; 78.0% were males; and 85.3% did not live with their parents or guardians. More than three quarters, 78.2% of the respondents were single while the rest were married. Regarding the level of education, more than half, 58.2% had attained post primary education while the remainder had only attained primary education or less. About 74.3% of the respondents had used alcohol in the last 30 days while more than half, 51.9% had used marijuana. (Table 1).

**Table 1:** Characteristics of young psychoactive substance users in the informal settlements of Kampala, Uganda

Variable	Attribute	Frequency (***n*** = 744)	Percentage (%)
Age category	18–19	174	23.4
	20–24	570	76.6
Sex	Male	580	78.0
	Female	164	22.0
Religion	Catholic	291	39.1
	Anglican	126	16.9
	Muslim	222	29.8
	Born again/ Pentecostal	80	10.8
	Other religion	25	3.4
Duration of staying in area (in years)	0–5	264	35.5
	6–10	145	19.5
	Greater than 10	335	45.0
Still staying with parents	Yes	109	14.7
	No	635	85.3
Average monthly income (Exchange rate: 1 USD = UGX 3755).	0–250,000	477	64.1
	250,001-500,000	204	27.4
	Above 500,000	63	8.5
**History of psychoactive substance use**			
Used alcohol in the last 30 days	Yes	553	74.3
	No	191	25.7
Used marijuana in the last 30 days	Yes	386	51.9
	No	358	48.1
Used khat in the last 30 days	Yes	409	55.0
	No	335	45.0
Used heroin in the last 30 days	Yes	14	4.0
	No	730	96.0
Used oral tobacco in the last 30 days	Yes	67	9.0
	No	677	91.0

*USD* United Staes Dollar*, UGX* Uganda Shillings

### Frequency of psychoactive substance use

**Fig. 1:**
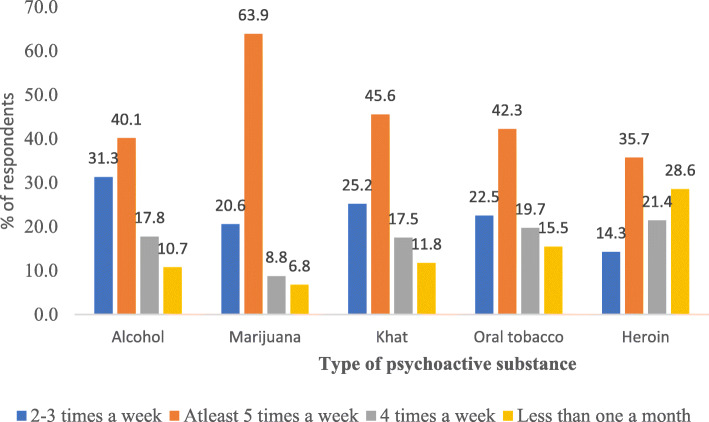
Frequency of psychoactive substance use in the last 30 days

About 40.1% of alcohol consumers used it at least 5 times a week; 63.9% of marijuana users used it at least 5 times a week; 45.6% of khat users used it at least 5 times a week while 42.3% of oral tobacco users used it at least 5 times a week. (Fig. 1).

### Sexual behaviours

The median age at sex debut was 16.0 years (IQR = 14, 17), with a median number of sexual partners in the last 12 months at 3 (IQR =1, 9). The median age at sex debut for males was 15.0 years (IQR = 14, 17) while that of females was 16 years (IQR = 14, 17). Nearly a quarter, 23.6% of respondents admitted ever intentionally intoxicating someone with psychoactive substances with the aim of luring them into sex. About 46.2% (43.8% of males and 54.8% of females) of respondents or their partners used a psychoactive substance before the last sexual intercourse. Among those who used psychoactive substances before sex, 55.6% (27.8% of males and 18.3% of females) mentioned that they alone had used the substances; in 39.2% (15% of males and 29.3% of females) both the respondent and the partner used the substances; in 5.2% (1% of males and 7.3% of females) only their sexual partner used the substances. Over half, 55.5% (27.4% of males and 19.5% of females) of the respondents who used the psychoactive substances before sex reported being intoxicated during the most recent sexual intercourse, 36.1% (13.4% of males and 28% of females) indicated that they and their partner were intoxicated during the last sexual intercourse and 5.5% (1.4% of males and 6.7% of females) said only their partner was intoxicated. Only 2.9% (1.5% of males and 0.6% of females) of respondents reported that none of them was intoxicated before the last sexual intercourse. About 36.5% of the 744 respondents had ever had sex with a commercial sex worker or sex client. About 36.8% of the males in the current study had ever had sex with a commercial sex worker while 35.4% of the females had ever had sex with a sex client. Out of the 744 respondents in our study, 32.5% had ever had sex with a commercial sex worker or client while intoxicated. About 41.5% of the females and 30% of the males had ever had sex with a sex client or commercial sex worker while intoxicated.

### Sexual expectancies and inhibitions

Over 45% of the respondents believed that psychoactive substance use improves sexual performance, 43.3% believed that sex is more pleasurable when under the influence of psychoactive substances, while more than half (53.1%) believed that using psychoactive substances gives courage or confidence to approach a partner for sex (Table 2).

**Table 2:** Sexual expectancies and inhibitions among young psychoactive substance users in the informal settlements of Kampala, Uganda

Variable	Attribute	Overall (***n =*** 744) (%)	Males n (%)	Females n (%)	***p-***value
Psychoactive substance use improves sexual performance	Yes	339 (45.6)	262 (45.2)	77 (46.9)	
	No	405 (54.4)	318 (54.8)	87 (53.1)	0.686
Sex is more pleasurable when under the influence of psychoactive substances	Yes	322 (43.3)	237 (40.9)	85 (51.8)	
	No	422 (56.7)	343 (59.1)	79 (48.2)	0.012*
Being under the influence of psychoactive substances make it difficult for you to use condoms	Yes	220 (29.6)	241 (40.0)	51 (30.9)	
	No	524 (70.4)	362 (60.0)	114 (69.1)	0.034*
Being under the influence of psychoactive substances make you forget to use a condom	Yes	205 (27.6)	163 (28.1)	42 (25.6)	
	No	539 (72.4)	417 (71.9)	122 (74.4)	0.528
Likely to engage in sex when under the influence of psychoactive substances	Yes	333 (44.8)	254 (43.8)	79 (48.2)	
	No	411 (55.2)	326 (56.2)	85 (51.8)	0.320
Likely to find it difficult to refuse sex when under the influence of alcohol or substances	Yes	274 (36.8)	217 (37.4)	57 (34.8)	
	No	470 (63.2)	363 (62.6)	107 (65.2)	0.533
Agreed that using psychoactive substances gives courage or confidence to approach a partner for sex	Yes	395 (53.1)	311 (53.6)	84 (51.2)	
	No	349 (46.9)	269 (46.4)	80 (48.8)	0.586
Find yourself wanting to have sex when using psychoactive drugs	Yes	352 (47.3)	270 (46.5)	82 (50.0)	
	No	392 (52.7)	310 (53.5)	82 (50.0)	0.435

### Association between sexual expectancies and inhibitions and high-risk sexual behaviours

Results in Tables 3 and 4 show that respondents who endorsed that they were likely to engage in sex when under the influence of psychoactive substances had a 48% higher likelihood of engaging in multiple sexual relationships in the last 30 days compared to those who did not think so (PR 1.48, 95% CI: 1.15–1.90). Respondents who indicated that it is difficult to refuse sex when under the influence of psychoactive substances had a 28% higher likelihood of engaging in multiple sexual relationships in the last 30 days compared to those who did not think so (PR 1.28, 95% CI: 1.06–1.55) (Table 3). Believing that there is a higher likelihood of forgetting to use a condom while under the influence of psychoactive substances was positively associated with inconsistent condom use in the last 30 days (PR 1.26, 95% CI: 1.09–1.45) (Table 4).

**Table 3:** Association between sexual expectancies and inhibitions, and multiple sexual partnerships among young psychoactive substance users in the informal settlements of Kampala, Uganda

Variable	Freq (n)	Engaged in multiple sexual relationships in the last 30 days		Unadjusted PR (95% CI)	***p-***value	Adjusted PR (95% CI)	***p-***value
		Yes	No				
**Age category (years)**							
18–19	174	73 (24.2)	101 (22.9)	1.0		1.0	
20–24	570	229 (75.8)	341 (77.1)	0.95 (0.78–1.17)	0.674	0.89 (0.73–1.07)	0.228
**Sex of the respondent**							
Male	580	220 (72.8)	360 (81.4)	1.0		1.0	
Female	164	82 (27.2)	82 (18.6)	1.31 (1.09–1.58)	0.003	1.31 (1.10–1.56)	0.003*
**Level of education**							
Primary and below	310	137 (45.4)	173 (39.1)	1.0		1.0	
Above primary	434	165 (54.6)	216 (60.9)	0.86 (0.72–1.02)	0.089	0.98 (0.83–1.16)	0.903
**Psychoactive substance use improves sexual performance**							
**No**	405	128 (42.4)	277 (62.7)	1.0		1.0	
Yes	339	174 (57.6)	165 (37.3)	1.62 (1.36–1.93)	**< 0.001**	1.13 (0.92–1.38)	0.225
**Psychoactive substance use makes sex more pleasurable**							
**No**	422	135 (44.7)	287 (64.9)	1.0		1.0	
Yes	322	167 (55.3)	155 (35.1)	1.62 (1.36–1.93)	**< 0.001**	0.99 (0.81–1.21)	0.975
**Psychoactive substance use makes it difficult to use condoms**							
Yes	220	112 (37.1)	108 (24.4)	1		1.0	
No	524	190 (62.9)	334 (75.6)	1.40 (1.18–1.66)	**< 0.001**	0.85 (0.70–1.04)	0.132
**Psychoactive substance use increases likelihood of forgetting to use a condom**							
No	539	193 (63.9)	346 (78.3)	1		1	
Yes	205	109 (63.1)	96 (21.7)	1.48 (1.25–1.76)	**< 0.001**	0.86 (0.71–1.06)	0.172
**Psychoactive substance use increases likelihood of engaging in sex**							
No	411	111 (36.8)	300 (67.9)	1.0		1.0	
Yes	333	191 (63.2)	142 (32.1)	2.12 (1.76–2.55)	**< 0.001**	1.48 (1.15–1.90)	0.002*
**Psychoactive substance use makes it difficult to refuse sex**							
No	470	153 (50.7)	317 (71.7)	1.0		1.0	
Yes	274	149 (49.3)	125 (28.3)	1.67 (1.41–1.97)	**< 0.001**	1.28 (1.06–1.55)	0.008*
**Psychoactive substance use gives courage/confidence to approach a partner for sex**							
No	349	103 (34.1)	246 (55.7)	1.0		1.0	
Yes	395	199 (65.9)	196 (44.3)	1.70 (1.41–2.06)	**< 0.001**	1.16 (0.93–1.45)	0.171
**Psychoactive substance use increases likelihood of wanting sex**							
No	392	107 (37.4)	285 (64.5)	1.0		1.0	
Yes	352	195 (64.6)	157 (35.5)	2.02 (1.68–2.44)	**< 0.001**	1.24 (0.96–1.61)	0.093

*CI* Confidence interval, *PR* Prevalence Ratio

**Table 4:** Association between sexual expectancies and inhibitions, and condom use among young psychoactive substance users in the informal settlements of Kampala, Uganda

Variable	Freq (n)	Condom use in the last 30 days		Unadjusted	***p***-value	Adjusted	***p***-value
		Consistent	Inconsistent	PR (95% CI)		PR (95% CI)	
**Age category (years)**							
18–19	174	76 (27.3)	98 (21.0)	1.0		1.0	
20–24	570	202 (72.3)	368 (79.0)	1.14 (0.99–1.32)	0.064	1.12 (0.97–1.29)	0.107
**Sex of the respondent**							
Male	580	223 (80.2)	357 (76.6)	1.0		1.0	
Female	164	55 (19.8)	109 (23.4)	1.07 (0.95–1.22)	0.234	1.09 (0.95–1.23)	0.185
**Level of education**							
Primary and below	310	115 (41.4)	195 (41.8)	1.0		1.0	
Above primary	434	163 (58.6)	271 (58.2)	0.99 (0.88–1.11)	0.898	1.05 (0.93–1.17)	0.387
**Psychoactive substance use improves sexual performance**							
**No**	405	157 (56.5)	248 (53.2)	1.0			
Yes	339	121 (43.5)	218 (46.8)	1.05 (0.93–1.17)	0.387		
**Psychoactive substance use makes sex more pleasurable**							
Yes	322	106 (38.1)	216 (46.4)	1.0		1.0	
**No**	422	172 (61.9)	250 (53.6)	1.13 (1.04–1.26)	**0.027**	1.00 (0.88–1.14)	0.931
**Psychoactive substance use makes it difficult to use condoms**							
Yes	220	55 (19.8)	165 (35.4)	1.0		1.0	
No	524	223 (80.2)	301 (64.6)	1.30 (1.17–1.45)	**< 0.001**	0.96 (0.83–1.10)	0.587
**Psychoactive substance use increases likelihood of forgetting to use a condom**							
No	539	230 (82.7)	309 (66.3)	1.0		1.0	
Yes	205	48 (17.3)	157 (33.7)	1.33 (1.20–1.48)	**< 0.001**	1.26 (1.09–1.45)	0.002*
**Psychoactive substance use increases likelihood of engaging in sex**							
No	411	175 (62.9)	236 (50.6)	1.0		1.0	
Yes	333	103 (37.1)	230 (49.4)	1.20 (1.07–1.34)	**0.001**	1.07 (0.92–1.25)	0.355
**Psychoactive substance use makes it difficult to refuse sex**							
No	470	193 (69.4)	277 (59.4)	1.0		1.0	
Yes	274	85 (30.6)	189 (40.6)	1.17 (1.04–1.30)	**0.005**	1.01 (0.89–1.15)	0.840
**Psychoactive substance use gives courage/confidence to approach a partner for sex**							
No	349	130 (46.8)	219 (47.0)	1.0			
Yes	395	148 (53.2)	247 (53.0)	0.99 (0.89–1.11)	0.951		
**Psychoactive substance use increases likelihood of wanting sex**							
No	392	164 (59.0)	228 (48.9)	1.0		1.0	
Yes	352	114 (41.0)	238 (51.1)	1.16 (1.04–1.29)	**0.008**	0.99 (0.85–1.16)	0.942

*CI* Confidence interval, *PR* Prevalence Ratio

The beliefs that psychoactive substance use improves sexual performance (PR 1.14, 95% CI: 1.01–1.30) or increases sexual urge (PR 1.20, 95% CI: 1.04–1.40) were associated with a higher likelihood of having sex while under the influence of psychoactive substances in the last 30 days. The feeling that using psychoactive substances gives courage or confidence to approach a partner increased the likelihood of having sex while under the influence of psychoactive substances in the last 30 days by 21% (PR 1.21, 95% CI: 1.05–1.39). Lastly, respondents who indicated finding themselves wanting to have sex when intoxicated had a 22% higher likelihood of having sex under the influence of psychoactive substances in the last 30 days compared to those who thought otherwise (PR 1.22, 95% CI: 1.03–1.43) (Table 5).

**Table 5:** Association between sexual expectancies and inhibitions, and ever had sex while under the influence of psychoactive substances among young psychoactive substance users in the informal settlements of Kampala, Uganda

Variable	Freq (n)	Had sex under the influence of drugs in the last 30 days		Unadjusted	***p***-value	Adjusted	***p***-value
		Yes	No	PR (95% CI)		PR (95% CI)	
**Age category (years)**							
18–19	174	82 (18.1)	92 (31.7)	1.0		1.0	
20–24	570	372 (81.9)	198 (68.3)	1.38 (1.17–1.68)	**< 0.001**	1.32 (1.13–1.54)	< **0.001**
**Sex of respondent**							
Male	580	337 (74.2)	243 (83.8)	1.0		1.0	
Female	164	117 (25.8)	47 (16.2)	1.22 (1.08–1.38)	**0.001**	1.19 (1.05–1.34)	0.004*
**Level of education**							
Primary and below	310	209 (46.0)	101 (34.8)	1.0		1.0	
Above primary	434	245 (54.0)	189 (65.2)	0.83 (0.74–0.93)	**0.002**	0.94 (0.84–0.15)	0.288
**Psychoactive substance use improves sexual performance**							
**No**	405	200 (44.1)	205 (70.7)	1.0		1.0	
Yes	339	254 (55.9)	85 (29.3)	1.51 (1.35–1.70)	**< 0.001**	1.14 (1.01–1.30)	0.029*
**Psychoactive substance use increases likelihood of forgetting to use a condom**							
No	539	294 (64.8)	245 (84.5)	1.0		1.0	
Yes	205	160 (35.2)	45 (15.5)	1.43 (1.28–1.59)	**< 0.001**	1.05 (0.93–1.18)	0.360
**Psychoactive substance use increases likelihood of engaging in sex**							
No	411	192 (42.3)	219 (75.5)	1.0		1.0	
Yes	333	262 (57.7)	71 (24.5)	1.68 (1.49–1.89)	**< 0.001**	1.20 (1.04–1.40)	0.013*
**Psychoactive substance use makes it difficult to refuse sex**							
No	470	254 (55.9)	216 (74.5)	1.0		1.0	
Yes	274	200 (44.1)	74 (25.5)	1.35 (1.20–1.50)	**< 0.001**	1.05 (0.93–1.18)	0.371
**Psychoactive substance use gives courage/confidence to approach a partner for sex**							
No	349	162 (35.7)	187 (64.5)	1.0		1.0	
Yes	395	292 (64.3)	103 (35.5)	1.59 (1.40–1.80)	**< 0.001**	1.21 (1.05–1.39)	0.006*
**Psychoactive substance use increases likelihood of wanting sex**							
No	392	178 (39.2)	214 (73.8)	1.0		1.0	
Yes	352	276 (60.8)	76 (26.2)	1.72 (1.52–1.95)	**< 0.001**	1.22 (1.03–1.43)	0.016*

*CI* Confidence interval, *PR* Prevalence Ratio

## Discussion

This is the first study to establish associations between sexual expectancies and inhibitions and high-risk sexual behaviours among young PSUs in the informal settlements of Kampala. Young PSUs in this study on average had an early sexual debut (15.2 years). The median age at sex debut was 15.0 years for females and 16.0 for females. These findings imply that young people using psychoactive substances, engage in early sexual intercourse compared to the national median age in the general population of 17.6 years for females and 18.0 years for males [64]. The differences in age at sex debut observed between females and males could be due to cultural norms, especially those encouraging more freedom and sexual experiences for boys than for girls [65, 66]. Cultural socialization has been reported to influence the sexual behaviours of boys and girls [67]. In Uganda boys have more autonomy and less parental restrictions compared to girls, which gives them a leeway to engage in sexual intercourse even at an early age [67, 68].

Our study revealed significant differences in the proportion of males and females who felt that being under the influence of psychoactive substances made it difficult for them to use condoms. A higher proportion of males found it difficult to use condoms, compared to females. This could be due to the varying effects of psychoactive substances on sexual behaviour. Some psychoactive substances may deprive male users, the ability to wear a condom while others may think that sexual intercourse is more pleasurable without condoms.

There is also evidence that some psychoactive substances could deprive male users of the ability to sustain an erection, hence difficulties in putting on a condom [69]. In addition, Calysn, et al. [70] in their study reported that men often pointed out the negative effects of condom use on sexual experience. Young PSUs in their study reported not feeling good or natural, condoms not fitting well, change in orgasm, interruption of the sexual mood, and interference with feelings close to one’s partner. A significantly higher proportion of females felt that sex was more pleasurable when under the influence of psychoactive substances. Although psychoactive substances have varying effects, it is believed that substances such as alcohol increase the sexual libido of women, and consequently pleasure [71, 72].

Nearly a quarter of the young PSUs in this study had ever made someone else intoxicated with psychoactive substances in order to have sex with them while nearly half of the young PSUs or their partners used a psychoactive substance before their last the sexual intercourse. This is because a considerable proportion of the young PSUs expect that the use of psychoactive substances would improve sexual performance, give courage or confidence to approaching a partner for sex, and make sexual intercourse more pleasurable as evidenced by the study findings. Similar findings have been reported in other studies [60, 73, 74]. Getting someone intoxicated would make it easier for the partner to engage in sexual activity against their consent, mainly due to impaired cognition and decision making [75, 76]. Using psychoactive substance prior to the last sexual intercourse was thought to improve their confidence to approach sexual partners. The use of these substances has been reported to increase aggression among users [77, 78]. These expectancies are likely to have lured some respondents into intoxicating their partners with psychoactive substances or even using psychoactive substances during their last sexual intercourse.

Respondents who felt that they were likely to engage in sex under the influence of psychoactive substances were more likely to engage in multiple sexual relationships compared to those who did not think so. Users of psychoactive substances are significantly more likely to have sexual intercourse compared to non-users [79]. Similarly, an increase in the sexual urge or libido, which often characterises psychoactive substance use, may have been an important driver for young people who find it difficult to refuse sex while under the influence of psychoactive substances for engaging in multiple sexual relationships. Having multiple sexual partners in Kampala’s informal settlements, a setting that already has a high prevalence of sex work and HIV among young people [46, 80] and the general population [81], is likely to escalate the transmission of sexually transmitted infections (STIs), including HIV/AIDS.

Respondents in our study, who felt they were likely to forget to use a condom, while under the influence of psychoactive substances, did not consistently use condoms. This is not surprising since psychoactive substances impair judgment and cognition [82]. Once under the influence of psychoactive substances, young people often fail to make the correct decisions or choices, including condom use. Our findings emphasize the need for risk reduction and other sexual and reproductive health interventions among this high-risk group.

This study also indicates that sexual expectancies such as improved sexual performance, confidence or courage, and increased sexual desire increased the likelihood of engaging in sex under the influence of psychoactive substances. Young PSUs often resort to the use of these substances as a way of sustaining erections and bettering sexual performance [60]. A significant proportion of young girls and women in the informal settlements of Kampala engage in commercial sex and use of psychoactive substances among female sex workers has been reported in several studies [80, 83, 84].

Young PSUs in this study were less sexually inhibited. Those who endorsed that they were likely to engage in sex when under the influence of psychoactive substances were more likely to have sex while intoxicated compared to those who did not think so. This too, could be attributed to the effects of psychoactive substance use of sexual urge, desire, and anticipated expectancies such as better sexual performance. There is evidence for instance that alcohol makes women more sensuous and more romantic [85]. It could be this feeling that propels young PSUs to further engage in sexual intercourse when intoxicated. Our findings should be an eye opener to policy makers and those who engage in HIV/AIDS programming on the immediate risk posed by young PSU as far as the transmission of HIV is concerned. It is therefore important to expand the HIV prevention services to integrate preventing and mitigating the effects of psychoactive substances and to stem the transition to injecting drug use, which is more complex to manage [36].

### Strengths and limitations of the study

To the best of our knowledge, this is the first study to establish an association between sexual expectancies and inhibitions and high-risk sexual behaviours among young PSUs in informal settlements. However, this was a cross-sectional design and thus, it was not possible to draw causal inferences between the influence of psychoactive substances and high-risk sexual behaviours. Sexual expectancies and inhibitions could be affected depending on the dose of the psychoactive substance used; therefore, these findings cannot be generalised to all PSUs with varied doses. Substance use and sexual behaviours were self-reported, which could be subject to both recall and social desirability biases. Besides, this study did not apply sample weights, making it prone to unequal selection probability [55, 86].

## Conclusion

This study has established the association between sexual expectancies and inhibitions with high-risk sexual behaviours i.e., inconsistent condom use, engagement in multiple sexual partnerships, and having sex while under the influence of psychoactive substances in informal settings where other complications like poverty are already exposing young people to STIs and HIV risk. Psychoactive substance use expectancies associated with high-risk sexual behaviours included the belief that psychoactive substances improve sexual performance and give courage or confidence to approach a sexual partner. Psychoactive substance use inhibitions associated with high-risk sexual behaviours included an increased likelihood of engaging in sexual intercourse, difficulties in refusing to engage in sexual intercourse, and forgetting to use condoms while intoxicated. The campaign against high-risk sexual behaviours should explicitly include a message that the use of psychoactive substances promotes high-risk sexual behaviours. Further research could focus on understanding how expectancies and inhibitions accrued from independent psychoactive substances influence high-risk sexual behaviours, and/or how the dose of psychoactive substances influences sexual expectancies and high-risk sexual behaviours.

## Supplementary Information

**Additional file 1.** Data collection tool.

## Data Availability

The datasets analysed during the current study are available from the corresponding author upon reasonable request.

## Abbreviations

AIDS: Acquired immunodeficiency syndrome
HIV: Human immunodeficiency virus
PR: Prevalence ratio
PSUs: Psychoactive substance users
RDS: Respondent Driven Sampling
STI: Sexually transmitted infection
