# Supplementary material for: Do sexual expectancies and inhibitions predict high-risk sexual behaviours? Evidence from a cross-sectional survey among young psychoactive substance users in informal settlements in Kampala, Uganda
Source: BMC Public Health. 2021 Aug 4;21:1506. doi: 10.1186/s12889-021-11536-8 (PMC8336356; doi:10.1186/s12889-021-11536-8)
Supplement: Supplementary file 1 — Additional file 1. Data collection tool. [file 12889_2021_11536_MOESM1_ESM.pdf]

Sexual behaviours of Young psychoactive substance users in Kampala

Sexual behaviours of young psychoactive substance users

Name of Data collector

Erinnya ly'omunonyereza

- Tonny
- Gloria
- Patience
- Ignatius
- Aisha
- Andrew
- Christine
- Job
- Olivia

In this study, I will ask you about the use of psychoactive substances such as khat, marijuana, alcohol, kabanga and heroine. In addition, I will ask you questions pertaining your sexual behaviour as well as information regarding sexually transmitted diseases such as syphilis.

Division

Divisoni

- Makindye
- Kawempe
- Rubaga
- Nakawa
- Central

Name of informal settlement

Erinnya ly'ekifo

- Kitintale
- Luzira-Kirombe
- Naguru-Go down
- Kinawataka
- Banda
- Luzira

Name of informal settlement

Erinnya ly'ekifo

- Katanga
- Mulago
- Kalerwe
- Bwaise
- Kyebando
- Kamwokya
- Kazo-Angola

Name of informal settlement

Erinnya ly'ekifo

- Nalukolongo
- Nalukolongo-Ssembuule
- Wankulukuku-Kabowa
- Ndeeba
- Nakulabye
- Kasubi
- Namugoona

Name of informal settlement

Erinnya ly'ekifo

- Kisenyi

Name of informal settlement

Erinnya ly'ekifo

- Namuwongo-Soweto
- Nabutiti-Kansanga
- Katwe-Kinyoro
- Kikuba mutwe-Kabalagala
- Kibuli
- Wabigalo
- Ggaba 1
- Ggaba II

GPS coordinates

latitude (x.y °)

longitude (x.y °)

altitude (m)

accuracy (m)

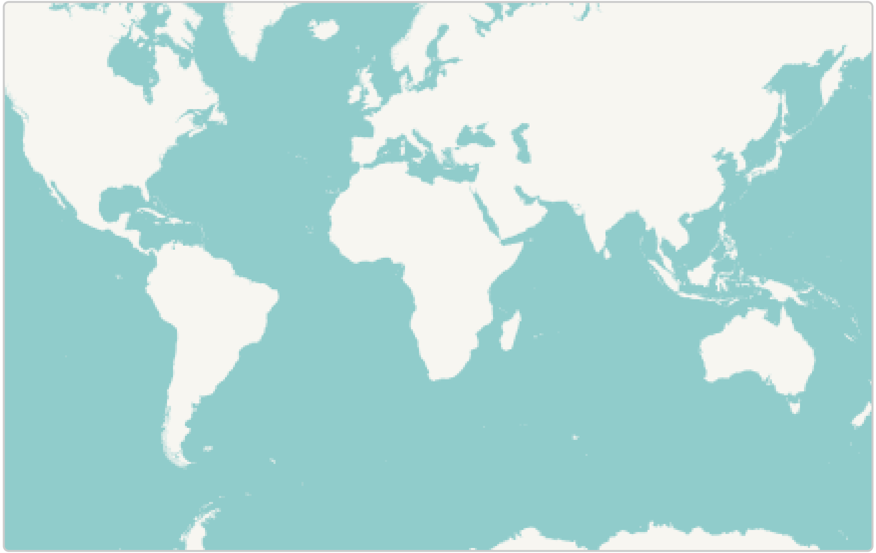

SECTION ONE: Socio demographic characteristics of the respondents

Ebikwata ku muntu abuuzibwa

Sex of the respondent

Ekikkula ky'omuntu

- Male
- Female

How old are you?

Olina emyaka emmeka emijjuvu

What is the highest level of school you attended/are in now?

Mubyokusoma, wakoma ku ddaala ki?

- No formal education
- Primary
- Secondary
- Tertiary

Marital status

Eby'obufumbo

- Single
- Married/ Cohabiting
- Widowed
- Divorced
- Separated

What is your religion?

Oli wa ddiini ki?

- Catholic
- Protestant
- Muslim
- Born again Christian/ Pentecostal
- SDA
- Other

For how long have you lived in this area? (Years) (If less than a year, go to next question)

Mukitundu kino abadde mu kumala emyaka emmeka?

____

For how long (Months) have you lived in this area?
Mukitundu kino obadde mu kumala emyezi emmeka?

____

What is your main source of income?
Okola murimu ki okwebezaawo?

- ◯ Fisherman/ Fish trader
- ◯ Petty Trader
- ◯ Bar Attendant
- ◯ Peasant/ Farmer
- ◯ Formal employment
- ◯ Sex work
- ◯ Drug dealer
- ◯ Other

Specify______________________________

Roughly how much do you earn per month? This may be from sales, part time work or other work
Bwogeregeranya ofuna ssente mmeka mumwezi?

____

Are you still staying with your parents?
Okyabeera ne bazadde bo?

- ◯ Yes
- ◯ No

HISTORY OF PSYCHOACTIVE SUBSTANCE USE

SECTION TWO: History of psychoactive substance use

The next few questions are about the use of PSYCHOACTIVE SUBSTANCES such as Beer, Waragi, Tonto, khat, marijuana, heroin and others by yourself and by people you know.

Ebibuuzo ebiddako by'ekuusa kukukozesa ebitamiiza nga ''beer'', omunanaansi, tonto, waragi, amalwa N'EBINTU ebirala nga enjaga, amairungi n'amafuta g'enyonyi.

INSTRUCTION 1: READ TO RESPONDENT: I am going to read to you some of the psychoactive substances commonly used in Uganda and I request you tell me whether you have ever used them or currently using them.Instruction: If the answer to the questions below is 'NO' write 'NA' for "not applicable" for next question then ask about the next drug or substance

Have you ever used alcohol (e.g Wines, spirits and beer)?

Wali Okozesezaako omwenge nga walagi, beer, tonto oba wine?

- Yes
- No

Have you used alcohol in the last 12 months?

Okozesezaako omwenge mu myezi 12 egiyise?

- Yes
- No

Have you used alcohol in the last 30 days?

Okozesezaako omwenge mu nnaku 30 eziyise?

- Yes
- No

During the last 30 days, how often did you use alcoholic substances such as beer, wines and spirits?

Munnaku asatu eziyise, mirundi emmeka gy'okozesa omwenge?

- Monthly or less (Includes daily consumption)
- 2 to 4 times a month
- 2 to 3 times a week
- 4 or more times a week

Are there times when any of the members of your social network (Friend, relatives, other) has condoned you to drink alcohol?

Wali wabaddewo akaseera nga mikwano gyo egy'okulusegere n'egikugaana okunywa omwenge?

- Yes
- No

Who condoned you? (Multiple response)

Ani yakugaana?

- Spouse
- Friends
- Children
- Relative/s
- Police
- Other

Specify the category of person

Have you ever used Marijuana/ Cannabis (Njaga/ sada)?

Wali okozesezaako enjaga/esada?

- Yes
- No

Have you used Marijuana/ Cannabis (Njaga/ sada) in the last 12 months?

Okozesezaako enjaga/ esada mu myezi 12 egiyise?

- Yes
- No

Have you used marijuana in the last 30 days?

Okozesezaako ku njaga mu nnaku 30 eziyise?

- Yes
- No

During the last 30 days, how often did you use marijuana/ canabis (njaga/ sada)?

Munnaku asatu eziyise, mirundi emmeka gy'okozesa enjaga?

- Monthly or less
- 2 to 4 times a month
- 2 to 3 times a week
- 4 or more times a week

Are there times when any of the members of your social network (Friend, relatives, other) has condoned you to use this substance?

Wali wabaddewo akaseera nga mikwano gyo egy'okulusegere negikugaana okunywa enjaga?

- Yes
- No

if yes, by whom? indicate whether (spouse, children, friends or other) (Multiple response)

- Spouse
- Friends
- Children
- Relative/s
- Police
- Other

Specify the category of person

Have you ever used khat (mairungi)?

Wali Okozesezaako amairungi?

- Yes
- No

Have you used khat (mairungi) in the last 12 months?

Okozesezaako amairungi mu myezi 12 egiyise?

- Yes
- No

Have you used khat it in the last 30 days?

Okozesezaako ko ku mairungi nnaku 30 eziyise?

- Yes
- No

During the last 30 days, how often did you use khat?

Munnaku asatu eziyise, mirundi emmeka gy'okozesa ko ku mairungi?

- Monthly or less
- 2 to 4 times a month
- 2 to 3 times a week
- 4 or more times a week

Are there times when any of the members of your social network (Friend, relatives, other) has condoned you to use khat?

Wali wabaddewo akaseera nga mikwano gyo egy'okulusegere n'egikugaana okulya amairungi?

- Yes
- No

if yes, who condoned you?

Muntu ki

- Spouse
- Friends
- Children
- Relative/s
- Police
- Other

Specify the category of person

Have you ever used kuba?

Wali Okozesezaako kuba?

- Yes
- No

Have you used kuba in the last 12 months?

Wali okozesezaako ko kuba mu myezi 12 egiyise?

- Yes
- No

Have you kuba in the last 30 days?

Okozesezaako ko kuba mu nnaku 30 eziyise?

- Yes
- No

During the last 30 days, how often did you use kuba?

Munnaku asatu eziyise, mirundi emmeka gy'okozesa kuba?

- Monthly or less
- 2 to 4 times a month
- 2 to 3 times a week
- 4 or more times a week

Are there times when any of the members of your social network (Friend, relatives, other) has condoned you to use kuba?

Wali wabaddewo akaseera nga mikwano gyo egy'okulusegere negikugaana okozesa kuba?

- Yes
- No

if yes, who condoned you? (Multiple response)

- [ ] Spouse
- [ ] Friends
- [ ] Children
- [ ] Relative/s
- [ ] Police
- [ ] Other
- [ ] Declined to respond

Specify the category of person

Muntu ki

Have you ever used Heroin (Mafuta g'enyonyi)?

Wali Okozesezaako?

- ( ) Yes
- ( ) No

Have you used Heroin (Mafuta g'enyonyi) in the last 12 months?

Okozesezaako a mafuta g'enyonyi mu myezi 12 egiyise?

- ( ) Yes
- ( ) No

Have you used Heroin (Mafuta g'enyonyi) in the last 30 days?

Okozesezaako a mafuta g'enyonyi mu nnaku 30 eziyise?

- ( ) Yes
- ( ) No

During the last 30 days, how often did you use Heroin (Mafuta g'enyonyi) ?

Munnaku asatu eziyise, mirundi emmeka gy'okozesa Mafuta g'enyonyi?

- Monthly or less
- 2 to 4 times a month
- 2 to 3 times a week
- 4 or more times a week

Are there times when any of the members of your social network (Friend, relatives, other) has condoned you to use Heroin (Mafuta g'enyonyi)?

Wali wabaddewo akaseera nga mikwano gyo egy'okulusegere negikugaana okozesa amafuta g'enyonyi?

- Yes
- No

If yes, by whom?

- Spouse
- Friends
- Children
- Relative/s
- Police
- Other
- Declined to respond

Specify____

Have you ever used Kabanga (mixture of tobacco and cannabis)?

Wali Okozesezaako Kabanga?

- Yes
- No

Have you used kabanga in the last 12 months?

Okozesezaako kabanga mu myezi 12 egiyise?

- Yes
- No

Have you used kabanga in the last 30 days?

Okozesezaako kabanga mu nnaku 30 eziyise?

- Yes
- No

During the last 30 days, how often did you use kabanga?

- Monthly or less
- 2 to 4 times a month
- 2 to 3 times a week
- 4 or more times a week

Do you at times inject your self with drugs?

Otera okwefumita empiso z'ebiragalalagala?

- Yes
- No
- Never

If yes, what drugs do you often inject your self with?

Information on sexual behaviour

Now, I want to ask you questions pertaining your sexual behaviour. Some of these questions may be sensitive but I would like to assure you that all the responses you give me will be treated with utmost confidentiality. I promise you that no one will get to know whatever we discuss. You may feel free not to answer those questions that may negatively impact on your mental health. However, answering those questions will be useful in thoroughly understanding the sexual behaviour of people who use psychoactive substances. Can I proceed with the interview? If yes, proceed with the interview and if No, terminate the interview

Have you ever had any sexual intercourse? (Sexual intercourse can be vaginal, oral or anal)

Wali wegaseko muby'omukwano?

- Yes
- No
- Declined to respond

How old were you when you first had sexual intercourse?

Walina emyaka emmeka wewasokera ddala okwegatta muby'omukwano?

Are there occassions when you had sexual intercourse with somebody when you are under the influence of drugs?

Wali wabaddewo akaseera wewegatta n'omuntu nga osindikiribwa omwenge oba ebiragalalagala?

- Never had any sexual Partner
- Never happened
- Rarely
- Often
- Always
- Declined to answer

How many times have you had sex in past one month? (Rounds) (Indicate 00 in case of NO SEX in the last one month)

Mirundi emmeka gy'ewegasse mu mwezi oguwedde?

How many sexual partners did you have in the past one month?
Mumwezi oguyise wegasse n'abantu bammeka?

____

How many sexual partners have you had in the past 12 months?
Atte mu myezi 12 egiyise, weggase n;abantu bammeka?

____

What main reason leads you to having more than one sexual- partner?

- [ ] For sexual satisfaction
- [ ] Money
- [ ] Peer pressure
- [ ] For pleasure
- [ ] Other

Specify____

Information on previous sexual partners

Now i want to ask you about the different sexual partners that you have had in the last one year. I know that these questions may be sensitive, however, your responses will be kept confidential

What is your relationship with the woman you last had sex? (For only males)

Wayina nkolagana ki n'omukazzi gwewasembayo okwegatta naye?

- Spouse
- Girl Friend
- Another Friend
- Casual Acquaintance
- Commercial Sex Worker
- Other (Specify)

Specify____

What is your relationship with the man you last had sex? (For only females)

- Spouse
- Boy friend
- Another Friend
- Casual Acquaintance
- Commercial Sex Worker
- Client (sex customer)
- Other (Specify)

Specify____

The last time you had sexual intercourse, did you or your partner drink alcohol or use any drug before sex?

Omulundi gwewasembayo okwegatta kugwe oba omwagalwawo wali okozesezaako ku kitamiiza oba ebiragalalagala?

- Yes
- No

Who used alcohol or the drug?

Ani yali akozeseza omwenge oba ebiragalalagala?

- Respondent Only
- Partner Only
- Respondent and Partner
- Declined to respond/ Don't remember

Were you or your partner drunk or ''high'' on any other substance before last sex?

Wemwegattira, gwe oba omwagalwawo kwaliko atamidde oba waliwo eyali akozeseza ebiragalalagala?

- Respondent Only
- No
- Respondent and Partner
- Declined to respond
- Partner Only

For how long have you had sexual relations with this partner? (Weeks) (Indicate 99 IF THE PARTICIPANT DECLINES TO RESPOND)

Omazze bbanga ki ngawegatta n'omuntu oyo?

____

How do you describe the level of condom use with this partner during the period of the relationship?

Mwakozesa kondomu bulikiseera oba nedda?

- All the time
- Only sometime
- Never

What was the main reason for not using a condom or inconsistently using it?

Nsonga ki eyabalemesa okukozesa kondomu obudde bwona?

- Respondent wanted a child
- Trusted partner
- Partner insisted not to use it
- Other
- Don't know

Specify____

Where did you get the condom for the last sex with this person?

- Shop
- Friend
- Health facility such as Clinic/Drug Shop/ Pharmacy
- Lodging house
- Market
- He/ She Had It
- Don't remember
- Other

Specify____

How long was it between first meeting that person and first having sex with the partner?
Kyakutwalira bbanga ki okuva wewasanga omuntu ono n'okwegatta naye?

- Within 24 hours
- Between 1 day and 1 week
- Between 1 week and 4 weeks
- Between 4 weeks and 6 months
- Between 6 Months and 1 year
- More than 1 Year

How did you meet your latest partner?

- Church
- Bar
- At school/ work
- Social event organized
- Through a friend
- Society, club or, interest group e.g rotary clubs
- Neighbours /Family friends
- Others (Specify)

Specify____

Perceptions on the influence of psychoactive substance use on sexual behaviour

Do you think alcohol or psycho active substance use improves your sexual performance?
Olowooza omwenge oba ebiragalalagala bikwonegeramu ammanyi mu by'okwegatta?

- Yes
- No

When drinking or using other substances, how often do you think about sex?

Bw'oba onnywa omwenge oba nga okozesa ebiragalalagala (erinnya okugeza njaga), mirundi emmeka gy'olowooza ku kwegatta?

- Always
- Sometimes
- Uncertain /unsure
- Never

Have you ever got a woman/man drunk/ given him/her drugs or alcohol in order to have sex?

Wali otamiziiza ko omuntu kubanga oba okumuwa ebiragalalagala nga oyagala mwegatte mumukwano?

- Yes
- No

Do you use a drinking/ drug use establishment (e.g a bar) as a place to meet sexual partners?

- Yes
- No

How often have you found it difficult to use a condom when you were drunk or had used drugs?

Mirundi emmeka gy'ofuna obuzibu okukozesa obupiira ng'otamidde oba nga okozeseza ebiragalalagala?

- Never
- Once only
- Occassionally
- Often
- Always

Have you ever found it difficult to use a condom when you were drunk or under the influence of a substance?

- Yes
- No

If you have ever found it difficult to use a condom when you were drunk, how often has this happened with a sex worker?

Bw'oba ofuna obuzibu okukozesa obupiira nga okozeseza ebiragalalagala, kino ky'akabaawo emirundi emmeka?

- Never
- Once only
- Occassionally
- Often
- Always

Are you more likely to engage in sex when under the influence of psychoactive substances?

Otera okwagala okwegatta nga okozeseza ebiragalalagala?

- Yes
- No

Do you think sex more pleasurable when under the influence of psychoactive substances?

Okwegatta kusinga kunyumira nga okozeseza ebiragalalagala?

- Yes
- No

Does being under the influence of psychoactive substances make it difficult for you to use condoms?

Bw'oba okozeseza ebiragalalagala kitera okuzibuwalira okukozesa obupiira?

- Yes
- No

Does being under the influence of psychoactive substances make you forget to use a condom?

Bw'oba nga onywedde omwenge oba ebiragalalagala, kitera okuretera okwerabira okozesa obupiira?

- Yes
- No

Do you find it difficult to refuse sex when under the influence of alcohol or substances?

Otera okukisanga mu obuzibu okugaana okwegatta nga okozeseza omwenge oba ebiragalalagala?

- Yes
- No

Do you use psychoactive substances to give yourself courage/confidence to approach a partner for sex?

Otera okunnywamu okufuna obuvumu okusaba omuntu akaboozi?

- Yes
- No

Do you find yourself wanting to have sex when using psychoactive drugs?

Otera okwagala okwegatta nga okozeseza ebiragalalagala?

- Yes
- No

Have you ever had sex with a commercial sex worker or sex client?

Wali wegaseko n'omuntu atunda akaboozi/ neeko?

- Yes
- No

Have you ever had drunk sex with a commercial sex worker? (Drunk also means being ''high'' on drugs)

Wali wegasse ko n'atunda akaboozi ng'a otamidde oba nga okozeseza ebiragalalagala?

- Yes
- No

Did you use a condom when having drunk sex with a commercial sex worker?

Wali okozesezako kondomu nga wegatta ne nneeko?

- Yes
- No

What was the main reason for not using a condom or inconsistently using it?

Nsonga ki eyabalemesa okukozesa kondomu obudde bwona

- Respondent wanted a child
- Trusted partner
- Partner insisted not to use it
- Other (specify)
- Don't Know

Specify______________________________

Have you had a non-spouse sexual partner in the past 30 days? (Besides a commercial sex worker)

Nga ogyeko Nneeko, wali wegaseko n'omuntu omulala atali mukyala wo mu by'omukwano?

- Yes
- No

The last time you had sex with a non-spouse partner, did you use a condom/s?

Wewasembayo okwegatta n'omuntu oyo, wakozesa akapiira?

- Yes
- No

What was the main reason for not using a condom or inconsistently using it?

Nsonga ki eyabalemesa okukozesa kondomu obudde bwona?

- Respondent wanted a child
- Trusted partner
- Partner insisted not to use it
- Other (specify)
- Don't know

Specify______________________________

Information on sexually transmitted diseases

Have you ever heard of any sexually transmitted diseases (STIs)?

Wali owuliddeko ku ndwadde z'obukaba?

- ○ Yes
- ○ No

What sexually transmitted diseases do you know? Tick all that apply

Ndwadde ki z'ewali owuliddeko?

- ☐ HIV/AIDS
- ☐ Syphilis (Kabotongo)
- ☐ Gonorrhea (Enziku)
- ☐ Chlamydia
- ☐ Genital warts
- ☐ Hepatitis B
- ☐ Other (specify)

Specify____

Have you suffered from syphilis in the last 12 months?

Olwadde ko kabotoongo mumyezi 12 egiyise?

- ○ Yes
- ○ No

Have you suffered from Gonorrhea in the last 12 months?

Olwadde ko enziku mumyezi 12 egiyise?

- ○ Yes
- ○ No

Have you suffered from Genital warts in the last 12 months?

Olwadde ko esnundo mubitundu by'ekyama mumyezi 12 egiyise?

- Yes
- No

Have you suffered from Hepatitis B in the last 12 months?

Olwadde ko ku hepatitis B buno mumyezi 12 egiyise?

- Yes
- No

Have you ever tested/ screened for HepatitisB?

- Yes
- No

What was the result?

- Positive
- Negative
- Declined to respond

Have you tested for HIV in the last 12 months?

- Yes
- No

What was the result?

- Positive
- Negative
- Declined to respond

Where did you test from?

- Health unit e.g. hospital, clinic, drug shop
- Community outreach program
- Home e.g. Self testing
- Other

Specify____

HEPATITIS B VACCINATION STATUS OF YOUNG PSYCHOACTIVE SUBSTANCE USERS

Now i want to ask you specific questions pertaining to hepatitis B vaccination. These questions will aim at understanding whether you have ever been vaccinated against HBV

Have you ever heard about hepatitis B vaccination?

- Yes
- No

How effective do you think hepatitis B vaccination is in protecting someone against hepatitis B virus infection?

- Not effective
- Slightly effective
- Very effective
- I don't know

Have you ever received hepatitis B vaccination?

- Yes
- No

Why have you not received hepatitis B vaccination? (Multiple responses allowed)

- [ ] I am not aware of hepatitis B vaccination
- [ ] I do not know where to go and receive it
- [ ] I don't have time
- [ ] It is expensive
- [ ] I don't see the need
- [ ] I am afraid of contracting the virus from the vaccine
- [ ] Not aware/ heard of hepatitis B disease
- [ ] Other (Specify)

Specify____

How many doses of hepatitis B vaccine have you received?

- ( ) 1 dose
- ( ) 2 doses
- ( ) 3 or more doses

When did you receive the last dose of hepatitis B vaccine?

- ( ) Less than one month ago
- ( ) 1-3 months
- ( ) 4-6 months ago
- ( ) More than 6 months ago

What do you think is the recommended full dose of hepatitis B vaccine?

- 1 dose
- 2 doses
- 3 or more doses
- I don't know

How long does a full dose of hepatitis B vaccine protect someone?

- Less than 1 year
- 1- 5 years
- 6-10 years
- 11-19 years
- 20 years and more
- I don't know

Thank you for your time
